# Evaluation of new generation macrolides for the treatment and metaphylaxis of contagious bovine pleuropneumonia (CBPP) in cattle experimentally infected with *Mycoplasma mycoides* subspecies *mycoides*

**DOI:** 10.1186/s12917-019-2197-x

**Published:** 2019-12-12

**Authors:** Geoffrey Muuka, Beatrice Otina, Hezron Wesonga, Benson Bowa, Nimmo Gicheru, Kristin Stuke, E. Jane Poole, Jeremy Salt, Angie Colston

**Affiliations:** 1 Central Veterinary Research Institute (CVRI), Ministry of Fisheries and Livestock, Lusaka, Zambia; 2 Faculty of Veterinary Medicine, University of Nairobi, Nairobi, Kenya; 3 Veterinary Science Research Institute (VSRI), Muguga, Kenya Agricultural & Livestock Research Organization, Nairobi, Kenya; 4 Global Alliance for Livestock Veterinary Medicines (GALVmed), Nairobi, Kenya; 5 International Livestock Research Institute (ILRI), Nairobi, Kenya; 6 Global Alliance for Livestock Veterinary Medicines (GALVmed), Edinburgh, UK

**Keywords:** *Mycoplasma mycoides*, Contagious Bovine Pleuropneumonia, CBPP, Antibiotic, Macrolide, Tulathromycin, Gamithromycin, Oxytetracycline, Lung Lesion, Africa

## Abstract

**Background:**

Contagious bovine pleuropneumonia (CBPP) caused by *Mycoplasma mycoides* subspecies *mycoides* (*Mmm*) is an important disease of cattle that causes serious economic losses. With the known effectiveness of new generation macrolides, tulathromycin and gamithromycin were assessed in comparison with oxytetracycline as a positive control and saline as a negative control for effectiveness in inhibiting lung lesion development, promoting resolution, preventing spread and bacteriological clearance in susceptible local cattle breeds in two separate studies in Kenya and Zambia. Animals were monitored for clinical signs, sero-conversion as well as detailed *post-mortem* examination for CBPP lesions.

**Results:**

Using the Hudson and Turner score for lesion type and size, tulathromycin protected 90%, gamithromycin 80%, and oxytetracycline 88% of treated animals in Kenya. In Zambia, all animals (100%) treated with macrolides were free of lung lesions, while oxytetracycline protected 77.5%. Using the mean adapted Hudson and Turner score, which includes clinical signs, *post-mortem* findings and serology, tulathromycin protected 82%, gamithromycin 56% and oxytetracycline 80% of the animals in Kenya whereas in Zambia, tulathromycin protected 98%, gamithromycin 94% and oxytetracycline 80%. The saline-treated groups had 93 and 92% lesions in Kenya and Zambia respectively, with *Mmm* recovered from 5/14 in Kenya and 10/13 animals in Zambia. Whereas the groups treated with macrolides were free from lesions in Zambia, in Kenya 5/15 tulathromycin-treated animals and 6/15 gamithromycin-treated animals showed lesions. Oxytetracycline-treated animals showed similarities with 3/14 and 4/15 showing lesions in Zambia and Kenya respectively and *Mmm* recovery from one animal in Kenya and six in Zambia. In both studies, lesion scores of saline-treated groups were significantly higher than those of the antibiotic treated groups (*p* < 0.001). In sentinel animals, CBPP lesions were detected and *Mmm* recovered from one and two animals mixed with the saline-treated groups in Kenya and Zambia respectively.

**Conclusions:**

This study demonstrated that tulathromycin, a mycoplasmacidal, can achieve metaphylactic protection of up to 80%, while non-recovery of *Mmm* from sentinels suggests macrolides effectiveness in preventing spread of *Mmm.* It is recommended that further studies are conducted to evaluate strategies comparing vaccination alone or combining vaccination and antibiotics to control or eradicate CBPP.

## Background

*Mycoplasma mycoides* subspecies *mycoides* (*Mmm*) is a mollicute bacteria that causes contagious bovine pleuropneumonia (CBPP), an infectious and contagious disease of cattle characterised by severe fibrinous exudative pneumonia [1]. The economic losses attributed to the disease are recognised as a major hindrance to livestock production in sub-Saharan Africa. The disease is associated with trade restrictions, cattle mortality and high morbidity leading to weakness, emaciation, reduced work ability and reduced fertility [2–4].

The disease has been successfully controlled in most developed countries [5] using cattle movement restrictions and stamping out policies in affected herds. However, in sub-Saharan Africa, the disease has continued to spread and affect new areas, mainly due to the uncontrolled movement of cattle associated with pastoral livelihood practices such as transhumance, communal grazing, congregation at markets, watering points, and night kraals [6]. CBPP control by vaccination has been adopted by most sub-Saharan African countries, but for numerous reasons a high percentage of the susceptible cattle population are not vaccinated. The main vaccine used is an attenuated live T1/44 vaccine strain that confers a short-term immunity, requiring booster vaccinations annually. With this vaccine, primal vaccination protects between 30 and 60% of the population [7], suggesting that infection can still occur in some herds considered vaccinated.

The inability to control the disease using vaccines only has precipitated discussions to include antibiotics in the management of CBPP outbreaks. Although the use of antibiotics is officially discouraged by many African countries, there is evidence of widespread use [8]. Several authors have reported on the benefits as well as shortcomings of antibiotics in the treatment of CBPP. Nicholas et al. reported to have successfully used danofloxacin to treat and prevent spread of CBPP in naturally infected herds [9]. Oxytetracycline, danofloxacin and tulathromycin demonstrated effectiveness in biological matrices as well as artificial media in inhibiting the growth of *Mmm*, thus proposing that the three could possibly be used in the treatment of CBPP [10]. These findings do not completely agree with those of Provost et al [1] who reported the inability of antibiotics to stop spread of the disease even though they alleviated clinical signs. Niang et al. observed sequestra formation following treatment with oxytetracycline and suggested the probability of a cure because the drug prevented evolution of the disease to chronicity in the treated group [11]. The persistence of viable *Mmm* in treated animals was reported in experimental infections recording losses due to CBPP [12], suggesting possible spread of the disease to susceptible animals. In a study by Huebschle et al, there was success in reducing mortalities due to CBPP as well as spread of the disease from naturally infected cattle using danofloxacin [13] but the drug failed to reduce clinical signs.

The studies reporting effectiveness of antibiotics for treatment and prevention of CBPP spread do not provide conclusive evidence that those antibiotics can be used to control the disease; raising the need for further research to enable informed decisions concerning the use of antibiotics in the treatment of CBPP. It is for this reason that studies were carried out in Kenya and Zambia, two countries where CBPP continues to have a great impact on livestock productivity. Since CBPP disease manifestation is known to show breed variation [1] and antimicrobials effectiveness may vary between strains in different countries, different cattle breeds and different challenge strains were used in assessing effectiveness of two new generation macrolides in inhibiting development or promoting resolution of lesions caused by *Mmm* and in preventing spread of the disease to susceptible cattle.

## Results

### Clinical signs and mortalities

Clinical responses in the saline-treated control animals (Group 1) at both study sites were typical of CBPP and included cough, respiratory distress and elevated rectal temperatures (rectal temperature ≥ 38.5 °C, determined as 1 °C above the upper confidence limit of the groups’ mean rectal temperature before in-contact exposure). During the first six weeks after treatment, Group 1 at both study sites had a significantly higher proportion of days with elevated temperature than each of the antibiotic-treated groups (*p* < 0.001 for all).

Overall, Group 1 in Kenya showed significantly (*p* < 0.05) higher scores for cough and respiratory distress and a significantly (*p* < 0.05) higher proportion of days with cough and respiratory distress than the treated groups. In Zambia, only the tulathromycin- and oxytetracycline-treated groups (Groups 2 and 4, respectively) had significantly (*p* < 0.05) lower respiratory distress scores and a lower proportion of days with respiratory distress compared to Group 1. The overall differences in scores for cough between the groups were not significantly different (*p* < 0.062) and the gamithromycin group did not show a significant difference in both parameters (cough and respiratory distress) when compared to control animals (Group 1). The sentinel sub-groups did not show significant differences in clinical observations or elevation of rectal temperatures. This is despite one sentinel animal in Kenya, co-mingled with the control animals (Group 1) showing elevated rectal temperatures for 26 days.

In the period following treatment, six Group 1 animals in Kenya and three in Zambia were euthanased due to CBPP; in addition one animal from Group 3 and one from Group 4 were euthanased in Kenya due to CBPP.

### Necropsy and lung lesion scores

Table 1 shows the lesion types observed at *post-mortem* examination, with 93% (*n* = 14/15) and 92% (*n* = 12/13) of animals in Group 1 in Kenya and Zambia, respectively, showing gross pathological lesions, characteristic of CBPP; including pleuritis, fibrinous and fibrous adhesions, hyperaemia, lung consolidation, necrosis as well as well-developed sequestra. Lung consolidation was characterised by hepatisation and marbling appearance and in some cases, the pleural cavity contained copious amounts of clear yellowish fluid. Out of those with lesions, 64% (*n* = 9/14) and 67% (*n* = 8/12) respectively, were of the chronic type.

**Table 1:** Lesion types observed at *post-mortem* examination

Study Site	Kenya (VSRI)					Zambia (CVRI)				
Treatment	Saline	Tulathromycin	Gamithromycin	Oxytetracycline	Sentinels	Saline	Tulathromycin	Gamithromycin	Oxytetracycline	Sentinels
Number of Cattle	15 (14^a^)	15	15	15	20	13	13	13	14	18
Number with lesions	14	5	6	4	1	12	0	2	3	2
Acute/Subacute % (n)	36 (5)	0	0	25 (1)	100 (1)^b^	33 (4)	0	0	33 (1)	100 (2)^b^
Chronic/Sequestra % (n)	64 (9)	60 (3)	67 (4)	0	0	67 (8)	0	0	67 (2)	0
Recovery % (n)^c^	0	40 (2)	33 (2)	75 (3)	0	0	0	100 (2)	0	0

^a^Tissue samples for one animal were missing, hence *n* = 14 for lesion scores. ^b^ Mingled with Group 1 (saline). ^c^ Previous lesions that are no longer active and thought to be unable to cause disease

In Kenya, the tulathromycin-treated (Group 2) animals showed lesions in 5/15 out of which 60% (*n* = 3/5) were of the chronic type. The gamithromycin-treated (Group 3) animals showed lesions in 6/15, with 67% (*n* = 4/6) showing the chronic type of lesions. The animals with lesions that were classified as neither chronic nor acute showed evidence of recovery from infection, characterized by fibrous tags on the visceral pleura. These were defined as lesions that were no longer active and therefore thought to be unable to cause disease and were observed in Groups 2, 3 and 4 in Kenya and in only Group 3 in Zambia.

The findings in Kenya contrasted with those in Zambia where none of the macrolide-treated Groups 2 (tulathromycin) and 3 (gamithromycin) animals showed evidence of CBPP lung lesions. However, there were similarities in the oxytetracycline-treated (Group 4) where n = 3/14 and 4/15 animals showed lesions in Zambia and Kenya respectively with one from each country showing acute/subacute CBPP lesions (Table 1) suggesting presence of active disease in this group.

Amongst the sentinels (Group 5), CBPP lesions were detected in one animal in Kenya and two animals in Zambia, mixed into the control group (Group 1). The disease was not detected in sentinel animals mixed with groups 2, 3 and 4. At both study sites, the average Hudson and Turner scores for lesion type and size were significantly higher in the control groups compared to the treated groups (*p* ≤ 0.05) (Table 2).

**Table 2:** Clinical, serological, pathological, and bacteriological parameters in treated animals

Study Site	Kenya (VSRI)				Zambia (CVRI)			
Treatment	Saline	Tulathromycin	Gamithromycin	Oxytetracycline	Saline	Tulathromycin	Gamithromycin	Oxytetracycline
Number of Cattle	15 (14^*^)	15	15	15	13	13	13	14
Max. number of pyrexia days	2	1	1	0	6	1	1	1
Number euthanased	6	0	1	1	3	0	0	0
Max. CFT titer	640 ^b^	320	320	80	2560	20	640	640
Number with acute/subacute or chronic lesions	14	3	4	1	12	0	0	3
Number (%) with *Mycoplasma* isolated from tissue, pleural fluid or BAL	5/14 (36%)	0	2/15 (13%)	1/15 (7%)	10/13 (77%)	1/13 (8%)	2/13 (15%)	6/14 (43%)
Number (%) with *Mycoplasma* isolated from nasal swabs	3/15 (20%)	1/15 (7%)	4/15 (27%)	3/15 (20%)	2/13 (15%)	0	2/13 (15%)	2/14 (14%)
Average HT lesion score (s.d.) ^a^	3.40 (2.03)	0.33 (0.72)	0.67 (1.18)	0.40 (1.55)	2.54 (1.90)	0	0	0.57 (1.22)
*p*-value for significance (lesion scores)	Groups (overall): *p* ≤ 0.001; s.e. 0.375				Groups (overall): *p =* 0.003; s.e. 0.438 (*n* = 13); 0.423 (*n* = 14)			
Comparison	Group 1 vs.	Group 2 *p* ≤ 0.001	Group 3 *p* ≤ 0.001	Group 4 *p* ≤ 0.001	Group 1 vs.	Group 2# *(p* ≤ 0.001)	Group 3# *(p* ≤ 0.001)	Group 4 *p* ≤ 0.003

^*^Tissue samples for one animal were missing, hence *n* = 14 for lesion score. ^#^Appropriate analysis only possible for data for Groups 1 and 4, as 2 and 3 were all negative. However, Groups 2 and 3 are considered to be significantly lower than Group 1 because the 95% *CI* for Group 1 > zero (approx. 1.5–3.5)

^a^ Lesion score calculated for the size and type of acute or chronic lung lesions. ^b^ At VSRI, samples indicating end-point titers beyond 1/640 were not titrated further, in which case the CFT titer was reported as 640

### Laboratory results

#### Serology

All animals were seronegative by CFT [20] when they entered the study. Prior to treatment, during the in-contact phase (co-mingling with intubates) 27% (*n* = 16/60) of the Group 1 to 4 animals in Kenya seroconverted by CFT, with the first seropositive animal recorded 35 days after contact. In Zambia 17% (*n* = 9/53) seroconverted by CFT with the first seropositive animal recorded 18 days after contact. Following treatment, 93% (14/15) of Group 1 animals in Kenya and 77% (10/13) in Zambia seroconverted by CFT while seroconversion by cELISA was 87% (13/15) in Kenya and 85% (11/13) in Zambia. Only 33% (5/15) of Group 2 (tulathromycin-treated) animals in Kenya and 8% (1/13) in Zambia seroconverted by both CFT and cELISA. The gamithromycin group showed some variation in the seropositive animals by CFT and cELISA in Zambia. Whereas 60% (9/15) of Group 3 (gamithromycin-treated) animals seroconverted by both CFT and cELISA in Kenya, in Zambia 23% (3/13) seroconverted by CFT and 8% (1/13) by cELISA. Forty percent (40%, 7/15) of animals in Group 4 (oxytetracycline- treated) in Kenya and 14% (2/14) in Zambia seroconverted by both CFT and cELISA. A summary of clinical, serological, and pathological parameters in treated and sentinel animals is shown in Tables 2 and 3, respectively.

**Table 3:** Clinical, serological, pathological, and bacteriological parameters in Sentinels

Study Site	Kenya (VSRI)				Zambia (CVRI)			
Treatment	Sentinel Saline	Sentinel Tulathromycin	Sentinel Gamithromycin	Sentinel Oxytetracycline	Sentinel Saline	Sentinel Tulathromycin	Sentinel Gamithromycin	Sentinel Oxytetracycline
Number of Cattle	5	5	5	5	5	4	4	5
Max. number of pyrexia days	15	0	0	0	0	0	0	0
Number euthanased	0	0	0	0	0	0	0	0
Max. CFT titer	640 ^b^	10	10	0	80	80	0	0
Number with acute/subacute or chronic lesions	1	0	0	0	2	0	0	0
Number (%) with *Mycoplasma* isolated from tissue, pleural fluid or BAL	1/5 (20%)	0	0	0	2/5 (40%)	0	0	0
Number (%) with *Mycoplasma* isolated from nasal swabs	0	0	0	0	0	0	0	1/5 (20%)
Average HT lesion score (s.d.)^a^	1.20 (2.68)	0	0	0	1.60 (2.19)	0	0	0

^a^ Lesion score calculated for the size and type of acute or chronic lung lesions. ^b^ At VSRI, samples indicating end-point titers beyond 1/640 were not titrated further, in which case the CFT titer was reported as 640

#### Mmm Culture

Cultivation of *Mmm* from lung, lymph nodes and pleural fluid led to recovery from 36% (*n* = 5/14) and 77% (*n* = 10/13) of Group 1 animals in Kenya and Zambia respectively. *Mmm* was not recovered from Group 2 (tulathromycin) in Kenya but was recovered from 8% (*n* = 1/13) in Zambia. In Group 3 (gamithromycin), *Mmm* was recovered from 13% (*n* = 2/15) in Kenya and 15% (*n* = 2/13) in Zambia. In Group 4 (oxytetracycline) *Mmm* was recovered from 7% (*n* = 1/15) in Kenya and 43% (*n* = 6/43) in Zambia. In the sentinel animals (Group 5), *Mmm* was recovered from 3/38 animals (1/20 in Kenya and 2/18 in Zambia), all three co-mingled with Group 1. *Mmm* was not recovered from sentinels co-mingled with other treatment groups.

From nasal swabs of both studies, *Mmm* was recovered from five animals in Groups 1 (saline) and 4 (oxytetracycline) and from six animals in Group 3 (gamithromycin) showing evidence of shedding *Mmm*. In comparison, only one Group 2 animal (tulathromycin) had *Mmm* recovered from nasal swabs. Despite one sentinel in Kenya showing clinical disease, none showed evidence of shedding *Mmm*, but in Zambia, *Mmm* was recovered in nasal swabs from one sentinel co-mingled with Group 4 (oxytetracycline). A summary of the *Mmm* isolation results from treated and sentinel animals is shown in Tables 2 and 3, respectively.

### Therapeutic efficacy

The percentage reduction in lung lesion scores (HT score for lesion type multiplied by the score for lesions size) brought about by treatment, was calculated as: % reduction = 100 * (Mean control group lung lesion score – Mean treatment group lung lesion score)/Mean control group lung lesion score. In the Kenyan study tulathromycin protected 90%, gamithromycin 80%, and oxytetracycline 88% of the animals from lung lesions. In the Zambian study, all animals (100%) treated with macrolides were free of lung lesions, whereas oxytetracycline treatment protected 77.5% of the animals from lung lesions.

Using the mean adapted Hudson and Turner score, which includes clinical signs, *post-mortem* findings and serology, in Kenya, tulathromycin protected 82%, gamithromycin protected 56% while tetracycline protected 80% of the animals. In Zambia, tulathromycin protected 98%, gamithromycin protected 94% while tetracycline protected 80% of the animals. HT scores for saline-treated (Group 1) animals were significantly higher than those for antibiotic-treated groups at both study sites (*p* ≤ 0.001). There was no significant difference between the macrolide-treated groups in either study site (*p* = 0.74 for both) (Table 4).

**Table 4:** Summary of adapted Hudson and Turner Scores in treated animals

Study Site	Kenya (VSRI)				Zambia (CVRI)			
Treatment	Saline	Tulathromycin	Gamithromycin	Oxytetracycline	Saline	Tulathromycin	Gamithromycin	Oxytetracycline
Number of Cattle	15 (14*)	15	15	15	13	13	13	14
Mean serology score (s.d.)	2.7 (0.8)	0.8 (1.2)	1.5 (1.4)	0.6 (0.8)	2.15 (1.28)	0.15 (0.55)	0.54 (1.05)	0.43 (1.09)
Range	0–3	0–3	0–3	0–2	0–3	0–2	0–3	0–3
Mean clinical score^a^ (s.d.)	1.1 (1.5)	0 (n/a)	0.2 (0.8)	0.2 (0.8)	0.77 (1.30)	0	0	0
Range	0–3	0	0–3	0–3	0–3	0	0	0
Mean post-mortem score^b^ (s.d.)	5.2 (4.6)	0.3 (0.7)	1.3 (2.7)	0.4 (1.6)	5.61 (3.62)	0	0	1.29 (2.67)
Range	0–12	0–2	0–9	0–6	0–12	0	0	0–8
Mean adapted Hudson & Turner Score (s.d.)	4.5 (2.5)	0.8 (1.2)	2.0 (2.1)	0.9 (1.0)	8.54 (4.35)	0.15 (0.55)	0.54 (1.05)	1.71 (3.69)
Range	0–8	0–3	0–7	0–3	0–15	0–2	0–3	0–11
*p*-value for significance (HT Score)	Groups (overall): *p* ≤ 0.001; s.e. 0.466				Groups (overall): *p* ≤ 0.001; s.e. 0.813 (*n* = 13); 0.783 (*n* = 14)			
Comparison	Group 1 vs.	Group 2 *p* ≤ 0.001	Group 3 *p* ≤ 0.001	Group 4 *p* ≤ 0.001	Group 1 vs	Group 2 *p* ≤ 0.001	Group 3 *p* ≤ 0.001	Group 4 *p* ≤ 0.001

^a^ Clinical score calculated as a combination of pyrexia and mortality; ^b^ Lesion score calculated as a combination of acute/subacute or chronic lung lesion (type and size) and tissue isolation of *Mycoplasma*. ^*^ Tissue samples for one animal were missing, hence *n* = 14 for PM and overall adapted HT score

A complete elimination of infection was not achieved. Regression analysis for both studies showed that the probability of presence of CBPP infection was significantly reduced in treated animals (*p* < 0.001).

In both studies, clinical signs and serological responses were observed in sentinel animals that were mixed with the saline-treated groups and in Zambia seroconversion was observed in sentinels that were mixed with tulathromycin-treated animals (Table 5).

**Table 5:** Summary of adapted Hudson and Turner Scores in Sentinels

Study Site	Kenya (VSRI)				Zambia (CVRI)			
Treatment	Sentinel Saline	Sentinel Tulathromycin	Sentinel Gamithromycin	Sentinel Oxytetracycline	Sentinel Saline	Sentinel Tulathromycin	Sentinel Gamithromycin	Sentinel Oxytetracycline
Number of Cattle	5	5	5	5	5	4	4	5
Mean serology score (s.d.)	0.6 (1.3)	0	0	0	0.80 (1.10)	0.75 (1.50)	0	0
Range	0–3	0	0	0	0–2	0–3	0	0
Mean clinical score^a^ (s.d.)	0.4 (0.9)	0	0	0	0	0	0	0
Range	0–2	0	0	0	0	0	0	0
Mean post-mortem score^b^ (s.d.)	2.4 (5.4)	0	0	0	3.20 (4.38)	0	0	0
Range	0–12	0	0	0	0–8	0	0	0
Mean adapted Hudson & Turner Score (s.d.)	1.4 (3.1)	0	0	0	4.00 (4.69)	0.75 (1.50)	0	0
Range	0–7	0	0	0	0–10	0–3	0	0

^a^ Clinical score calculated as a combination of pyrexia and mortality; ^b^ Lesion score calculated as a combination of acute/subacute or chronic lung lesions (type and size) and tissue isolation of *Mycoplasma*

## Discussion

In order to evaluate the therapeutic efficacy of antibiotics against CBPP, a reliable experimental challenge mimicking natural infection is required. Experiments carried out at VSRI in Kenya and CVRI in Zambia showed that a 1:1 ratio of intubation (infected) animals to susceptible in-contact animals as an experimental infection model successfully led to transmission of the disease with similar pathological outcomes to natural transmission. Although this model differs with natural infections in terms of the high number of infected animals available, it has demonstrated the ability to transmit disease, as seen in earlier studies. The infection of controls was 93 and 92% in Kenya and Zambia respectively, demonstrating the effective spread of the disease from infected intubates to the susceptible group. The results of these studies therefore are in agreement with results of earlier studies and the model used here remains the preferred route of artificial infection until a less intrusive model demonstrates similar results.

The use of antibiotics against CBPP has long been controversial, though some earlier studies showed evidence suggesting efficacy in preventing transmission of the disease [13], largely preventing formation of sequestra, which is believed to be the primary source of carrier status [11] and reduction in clinical cases and death after treatment [9, 14]. There have also been some studies and pronouncements that have supported the belief that antibiotics lead to chronic carriers [1, 14] and found no evidence that antibiotic treatment reduced case fatality risk and average duration of clinical signs, although the authors speculated that the most widely used medication, a single intramuscular injection of 10–20 ml of 10% oxytetracycline suspension, was unlikely to achieve recovery [15] and has little impact on outcome of clinical cases [15]. Although the drugs would promote clinical alleviation of pain and discomfort in cattle and reduce excretion of *Mmm* and thus reduce new infections, the need to evaluate the development of antibiotic resistance cannot be overemphasised.

Carrier animals pose the most difficult challenge in the control and eradication of CBPP [1]. Any intervention that would reduce or curtail the development of carrier animals would be the most effective way of controlling and eradicating CBPP. Niang et al. reported that oxytetracycline largely prevented sequestra formation under experimental conditions demonstrating the probability of cure without evolution of the disease to chronicity to the treated group [11].

In this study the lack of acute or subacute lesions, which usually has the symptomatic clinical phase of the disease, in the treated groups, signifies effective action of the drugs against *Mmm*. This attribute would be useful in relieving the clinical signs of the diseases usually associated with pain in affected cattle in the field thus reducing the suffering of animals especially in the clinical phase of the disease. However, at both sites out of all Group 4 (oxytetracycline) animals with lesions, 25 and 33% showed acute lesions in Kenya and Zambia, respectively. This could mean that oxytetracycline either takes slightly longer to clear the *Mmm* during infection or may not clear the infection at all. Sentinel cattle (Group 5) from both sites that were mixed with antibiotic treated groups did not develop any pathological signs of CBPP while those that were mixed with the control group developed lesions that were acute/subacute at *post-mortem* (Table 5). This shows that the animals in the control group were still actively shedding adequate *Mmm* to infect the cattle in contact while those in the antibiotic group could either be free from or have fewer *Mmm* excreted to cause lesions at the point of examination. Analysis of the lesions showed that most of the chronic lesions consisted of pleuritis and extensive fibrosis or small, encapsulated sequestra; suggesting that there is little risk for healthy in contact cattle. This finding could prove very significant in curtailing the transmission of CBPP to susceptible cattle following antibiotic administration. Since *Mmm* is transmitted to susceptible animals in close contact by droplets emanating from either cattle with clinical disease or subclinical carriers that are actively excreting the organism [1, 16], the findings of this study strengthen the hypothesis that antibiotics could have reduced the quantities of the *Mmm* excreted at the time point of *post-mortem* examination hence the cattle not becoming infected with overt clinical signs. While out of all the animals with lesions in Group 2 (tulathromycin), 40% (n=2) had recovery and 60% (n=3) had chronic lesions in the Kenyan study, the similar group in Zambia did not have any lesions at all. This is indicative of the superior effectiveness of this drug (tulathromycin) compared to the others.

Recovery of *Mmm* from lesions demonstrates the invasive nature of the pathogen during infection. In this study, the recovery of *Mmm* was higher in the saline control groups than in the antibiotic-treated groups. Altogether, one tulathromycin-, four gamithromycin-, and five oxytetracycline-treated animals were positive for *Mmm* by isolation. The recovery of *Mmm* demonstrates that even though clinical recovery and lesion development was significantly inhibited through antibiotic treatment, bacteriological clearance of the *Mmm* was not complete. However, it is not known if the sentinel cattle introduced later played a role in maintaining the pathogen in the groups.

## Conclusion

This study has demonstrated that tulathromycin, gamithromycin and oxytetracycline significantly reduced clinical signs, mortality, *post-mortem* lesions and spread to susceptible cattle. Further evaluation is required to identify optimum strategies for using antibiotics alone or a combination of treatment and vaccination that could control or lead to eradication of the disease.

## Methods

### Study sites and ethical considerations

In 2016, a study was initiated at the Veterinary Sciences Research Institute (VSRI) of the Kenya Agricultural Research Institute (KALRO) in Muguga in Kenya followed by a study in 2017 at the Central Veterinary Research Institute (CVRI) in Balmoral in Zambia.

All animals were handled in compliance with applicable animal welfare regulations including safe disposal of animals. At VSRI, the study protocol was reviewed and approved by the Animal Care and Use Committee of the Veterinary Science Research Institute - Kenya Agricultural & Livestock Research Organization (approval code: KALRO-VSRI/IACUC010/07102016). At CVRI, the study protocol was approved by the Ministry of Agriculture Ethics Committee (approval number 2014/001).

### Experimental animals and acclimatisation

In both Zambia and Kenya, cattle were sourced from known CBPP free areas. In Zambia, a total of 130 male crossbreed *Bos indicus* x *Bos taurus* cattle (one to two years of age, weighing 126–257 kg) were acquired from private sources (Mighty Farms, Namwala District in the Southern Province of Zambia). In Kenya, a total of 146 male small East African zebu (*Bos indicus*) cattle (two to four years of age, weighing 104–215 kg) were sourced from small scale farmers through the local market and assembled at a local research station for one-month quarantine in Kakamega County in western Kenya, before transportation to the study site. At both study sites, the cattle were acclimatised to the local environmental conditions and husbandry systems for seven weeks. After purchase, the animals were ear-tagged and confirmed seronegative for CBPP antibodies using the complement fixation test (CFT). In addition, they were tested for trypanosomiasis, foot and mouth disease, bovine tuberculosis, with additional tests for East Coast fever conducted at CVRI. At VSRI, the cattle were vaccinated against lumpy skin disease and foot and mouth disease, while at CVRI, the cattle were vaccinated against pasteurellosis, anthrax and blackleg infection. They were also treated for external and internal parasites. The animals received good quality hay and had access to water and mineral bricks *ad libitum*. No animals were treated with any antibiotic compounds during acclimatisation before the study commenced.

### Experimental design

After acclimatisation, animals were randomly allocated to Group 0 (intubates, VSRI: *n* = 60; CVRI: *n* = 50) and Group 5 (sentinels, *n* = 20). Groups 0 and 5 were then group-housed in two separate pens that were at least 50 m apart, while the remaining combined group of animals (VSRI: n = 66; CVRI: *n* = 60) stayed in their original housing (this group is later referred to as the in-contact animals). On Day 0 of the study, Group 0 animals were infected by intubation. When 12% of intubates had indicative signs of CBPP (cough, depression, respiratory distress and/or pyrexia), the healthy in-contact animals were co-mingled with the intubates, sharing the same airspace in a 1:1 ratio. After co-mingling, all animals at VSRI were housed continuously indoors for one week to ensure close contact. Thereafter, they were housed indoors only at night. At CVRI, animals were continuously kept together outdoors. All animals were observed daily until at least 12 of the in-contact cattle showed signs suggestive of CBPP. At VSRI, the group of Afadé-infected in-contact animals showed signs suggestive of CBPP 42 days after exposure. At CVRI, signs suggestive of CBPP were seen within the group of Caprivi-infected in-contact animals 39 days after exposure.

Following confirmation of occurrence of CBPP, the in-contact cattle (VSRI: *n* = 60; CVRI: *n* = 53) were randomly allocated into four groups and treated with either saline as a negative control group (Group 1), tulathromycin (Group 2), gamithromycin (Group 3), or oxytetracycline as positive control (Group 4). The randomisation was balanced based on clinical symptoms and rectal temperature, so that an equal number of animals showing CBPP symptoms were included in the various groups. Groups 1 to 4 were group-housed by treatment group in pens situated at least 20 m apart from each other. Before the start of the treatment phase, one in-contact animal at VSRI and seven in-contact animals and one sentinel at CVRI were euthanased on ethical grounds unrelated to CBPP and then necropsied to establish their cause of death. Euthanasia was performed by stunning using a captive bolt pistol and exsanguination. Before treatment allocation, five clinically normal in-contact animals at VSRI were randomly dropped from the study as spares and remained at VSRI for research purposes outside the scope of this study. For purposes of this study, Group 0 (intubates) completed the study on the day of treatment with test products. By then 22 of the Group 0 animals at VSRI had been removed from the study for welfare reasons when they developed symptoms suggestive of severe CBPP. These animals were stunned, exsanguinated and necropsied to confirm their cause of death. All remaining Group 0 animals (intubates) at VSRI that were still alive (*n* = 38) were released from the study and remained at VSRI for research purposes outside the scope of this study. At CVRI, 36 Group 0 animals were stunned, exsanguinated and necropsied when they completed the study. Fourteen animals of this group were removed before they completed the study for welfare reasons and either necropsied after being found dead or euthanased by stunning and exsanguination and then necropsied to determine the cause of death.

One month after treatment, sentinel animals (Group 5) were co-mingled into each of the four treatment groups, at a ratio of 1:3. Monitoring continued for 61 days at VSRI and for 46 days at CVRI. After co-mingling one sentinel animal at CVRI was removed from the study after it was found dead for reasons unrelated to CBPP.

All Group 1 to 4 animals that entered the treatment phase (VSRI: *n* = 60; CVRI: *n* = 53) and all Group 5 animals that were co-mingled with treatment groups (VSRI: *n* = 20; CVRI: *n* = 18) were stunned, exsanguinated and *post-mortem* examinations were carried out from days 92–103 and 73–77 after treatment at VSRI and CVRI, respectively, unless euthanasia by stunning and exsanguination was required before the end of the study for welfare reasons. No study animal entered the human food chain. The carcasses of all animals were disposed of at VSRI and CVRI using deep lime pits.

The stages of lung lesion development and the lesion sizes were recorded and assigned a score according to the method described by Huebschle et al [13]; adapted from Hudson and Turner (1963). The number of cattle per group and the duration of the experiment was slightly different between the two study sites. The differences and similarities between both studies are summarised in Table 6.

**Table 6:** Experimental design and summary of study treatment groups

Group	Treatment	Dosage (mg/kg), Regimen, Route	CBPP Infection Kenya (VSRI)/Zambia (CVRI)	Number of Animals Kenya (VSRI)/Zambia (CVRI)^a^	Intubation	Start of In-Contact Exposure Kenya (VSRI)/Zambia (CVRI)	Treatment Administration Kenya (VSRI)/ Zambia (CVRI)	Observation Period Kenya (VSRI)/Zambia (CVRI)
0	None	n/a	Intubation with Afadé/Caprivi strain	60/50	Day 0	8/11 days after intubation	n/a	49/49 days after intubation
1	Saline	6.0 mg/kg Once SC	In-contact with Group 0	15/13 (15)	n/a	8/11 days after intubation	41/38 days after start of in-contact exposure	92/73 days after treatment
2	Tulathromycin	2.5 mg/kg Once SC	In-contact with Group 0	15/13 (15)	n/a	8/11 days after intubation	41/38 days after start of in-contact exposure	92/73 days after treatment
3	Gamithromycin	6.0 mg/kg Once SC	In-contact with Group 0	15/13 (15)	n/a	8/11 days after intubation	41/38 days after start of in-contact exposure	92/73 days after treatment
4	Oxytetracycline	20.0 mg/kg Once IM	In-contact with Group 0	15/14 (15)	n/a	8/11 days after intubation	41/38 days after start of in-contact exposure	92/73 days after treatment
5	None	n/a	In-contact with Groups 1–4	20/18 (20)	n/a	32/28 days after treatment (Groups 1 to 4)	n/a	60/45 days after co-mingling

^a^Numbers in brackets indicate the number of animals originally included into the study at CVRI: Seven animals from the combined group were withdrawn for welfare reasons unrelated to CBPP, before the start of the treatment phase and one sentinel was withdrawn before and one after co-mingling for welfare reasons unrelated to CBPP

To reduce bias, treatments were dispensed and administered by personnel who were not otherwise involved in making any clinical assessment. The veterinarians who assessed the animals for clinical signs of CBPP and *post-mortem* examinations and the personnel involved in laboratory testing were unaware of treatment allocation.

For purposes of statistical analysis, the individual animal was regarded as the experimental unit. Whilst co-mingling of treatment groups would have been preferable from a statistical point of view, this was not possible due to the chance for cross-infection. Therefore, to reduce variability, the management and environment (e.g. the structure of enclosures and ambient temperatures) of the animals were made as identical as possible and the study procedures were performed contemporaneously.

A 2-sample 2-sided binomial sample size calculation with normal approximation was used to estimate the number of animals required per group to compare the negative control treatment (Group 1) to any of the treatment groups (Group 2–4) with presence or absence of lung lesions as the outcome variable, hypothesised to be 50% for the negative control group and as low as 2.5% for any of the treatment groups. The sample size was calculated with 80% power and at the 5% level of significance. The required number of animals was n = 13 which was rounded up to 15 to account for drop-outs.

### Challenge

At both sites, infection of the Group 0 cattle with pathogenic *Mmm* cultures was carried out with an intubation tube inserted through the nostril of an animal until the tube reached the bifurcation of the trachea. At VSRI, cultures were obtained from the Afadé strain of *Mmm* [17] earlier donated to International Livestock Research Institute (ILRI) in Kenya by CIRAD (French Agricultural Research Centre for International Development). The Afadé strain originated from northern Cameroon and was isolated at the Farcha laboratories in Tchad in 1965. At CVRI, cultures were obtained from the Namibian Caprivi Strip *Mmm* strain outbreak of 1994 provided by the Instituto Zooprofilactico Sperimantale (IZS) of Teramo in Italy. The strains were cultured at 37 °C in Gourlay medium and harvested in the log phase of growth [18]. The number of organisms was determined by micro-titration in liquid media [19] and colonies were screened for the typical fried egg appearance of *Mmm*.

The cultures were pooled and aliquoted in vials of 60 mL each for VSRI, while at CVRI, they were aliquoted into 40 mL vials, and at both sites, stored at − 80 °C to provide a standardised source of inoculum as described elsewhere [17]. At VRSI, each Group 0 animal received 60 mL of *Mmm* culture containing 8.6 × 10^7^ colony forming units (cfu)/mL, followed sequentially by 30 mL of 1.5% agar solution and 30 mL of PBS. At CVRI, each Group 0 animal received 30 mL of *Mmm* culture containing 10^9^ cfu/mL, followed sequentially by 20 mL of sterile 2% agarose gel and 20 mL of PPLO broth [18]. The challenge titer was defined as the mean titer of the pre- and post-challenge samples.

### Treatments

A commercial formulation of tulathromycin 10% injection (Draxxin™, Zoetis) was administered at 2.5 mg/kg. A commercial formulation of gamithromycin 150 mg/ml solution for injection (Zactran™, Merial) was administered at 6 mg/kg. A commercial formulation of oxytetracycline was administered at 20 mg/kg (VSRI: Alamycin™ LA 20, Norbrook Laboratories; CVRI: Oxy™ 20% LA, Dopharma). Physiologically normal saline was administered at 6 mg/kg to provide an equivalent volume for injection. The macrolides and saline were administered via a single sub-cutaneous (SC) injection in the side of the neck. Oxytetracycline was given intramuscularly (IM) in the left hindquarter.

### Clinical and post-mortem examinations

Clinical parameters were assessed throughout the study. After intubation, animals in Group 0 were checked daily for rectal temperature and signs suggestive of CBPP, i.e. cough, depression, respiratory distress, nasal discharge, general condition and appetite. In addition, blood and nasal swab samples were collected from all intubates at weekly intervals. Signs of CBPP in Group 0 were documented eight (at VSRI) and 11 (at CVRI) days after intubation.

Following co-mingling and subsequent treatment of the in-contact animals (Groups 1 to 4) the animals were checked daily for clinical signs of CBPP and rectal temperatures; blood samples were collected bi-weekly and nasal swabs weekly; the last samples were collected on the day of necropsy. Signs of cough, depression, respiratory distress, nasal discharge, general condition and appetite were categorised as absent (score 0), mild (score 1), moderate (score 2), or severe (score 3).

Any animal with cough, depression or respiratory distress categorised as severe was withdrawn, euthanased and examined at *post-mortem* to confirm CBPP-related lesions. Samples for bacteriological examination and PCR were collected from these animals.

### Laboratory analysis

Nasal swab samples collected during the study were agitated in three mL of PPLO broth (at VSRI) or in four mL of PBS (at CVRI), cultured in Newings Tryptose [20] and stored frozen at or below − 20 °C in media until analysed. Nasal swab samples from both study sites, collected from Groups 1 to 4 just before treatment, and two and four weeks after treatment, were shipped to CVRI’s Microbiology and Molecular Biology Laboratory where they were processed as described in the OIE Manual [20].

*Post-mortem* tissue and fluid samples including peribroncheal lymph nodes, caudal mediastinal thoracic lymph nodes, lung tissue (including a sample from a representative lesion, where present), pericardial fluid, joint synovial fluid, pleural fluid, kidneys, and bronchio-alveolar lavage (BAL, only on lungs without visible lesions) were collected from any animal that died or was euthanased. Samples were stored frozen at or below − 20 °C until analysed. Microbiological culturing was done by the respective Microbiology and Molecular Biology laboratories at VSRI and CVRI for all Groups 1 to 5 animals. Isolates from VSRI were shipped for molecular diagnosis to the Department of Bacteriology of the Animal and Plant Health Agency (APHA) in the UK and identified using *Mmm* specific PCR described by Bashirudin et al [21]. CVRI’s culture positive isolates were tested by *Mmm* specific PCR-DGGE [22] at its Microbiology and Molecular Biology Laboratory.

### Serology

Serum was separated and stored frozen at or below − 20 °C before analysis for CBPP antibodies by CFT and c-ELISA. The CFT was conducted according to the procedure described in the OIE Manual [20]. End-point titers were the highest dilutions of sera producing 50% hemolysis/inhibition of sheep red blood cells, given as reciprocals of these dilutions. At VSRI, samples indicating end-point titers beyond 1/640 were not titrated further, in which case the CFT titer was reported as 640. At CVRI, samples were titrated to the end-point. The c-ELISA assay was conducted and interpreted according to c-ELISA test kit instructions (Idexx®).

### Statistical analysis

Data were summarised and analysed using Genstat® [23], comparing the pre-and post- treatment phases. Efficacy was based on lung lesion scoring with reduction in lung lesion scores brought about by treatment, as well as on evaluation using the adapted Hudson and Turner Score by Huebschle et al. This includes combining clinical responses based on pyrexia and CBPP-related mortalities, evaluation of *post-mortem* lung lesions, *Mmm* isolation, and serological responses by CFT [13], to obtain a single score per treatment group. Analysis for CBPP antibodies by c-ELISA and *Mmm* isolation from nasal swab samples were secondary variables.

**Table:** 

Adapted Hudson and Turner Score:	
Serological response (CFT) to challenge	
< 10	Score 0
10	Score 1
20–80	Score 2
= or > 160	Score 3
Clinical response to challenge	
Pyrexia at least (≥) 39.5 °C for > 3 but < 7 days	Score 1
Pyrexia for ≥7 days	Score 2
Animal died/euthanased	Score 3
*Post-mortem* findings	
Chronic lesions (sequestra)	Score 1
Acute/subacute (marbling etc.)	Score 2
*Mmm* isolated or detected	Score 2
Multiply the sum by a factor depending on lesion size:	
< 5 cm	Score 1
≥ 5 cm but < 20 cm	Score 2
≥20	Score 3
Thus, maximum possible score is 18:	
Serology	= maximum possible score is 3
Clinical	= maximum possible score is 3
PM	= (2 + 2) 3 = 12

The adapted Hudson and Turner score and individual lung lesion scores of the treatment groups were used separately in the analysis of variance (ANOVA) and no transformation was required. The differences between each of the treatments (2, 3 and 4) vs. the saline-treated controls (1) were estimated with 95% confidence intervals, with significance tests based on linear contrasts between the means. The same ANOVA methodology was used to analyse elevated temperatures (average temperature and proportion of days with elevated temperature) and clinical observations (average score and proportion of days with clinical sign).

The probability of finding CBPP lesions was analysed using logistic regression with t-tests used to compare the differences, on the linear scale, between treatments (2, 3 and 4) vs. the saline-treated controls (1).

## Data Availability

The datasets used and/or analysed during the current study are available from the corresponding author on reasonable request.

## Abbreviations

ANOVA: Analysis of variance
APHA: Animal and Plant Health Agency
BAL: Bronchio-Alveolar Lavage
BMGF: Bill & Melinda Gates Foundation
CBPP: Contagious Bovine Pleuropneumonia
c-ELISA: Competitive Enzyme Linked Immunosorbent Assay
CFT: Complement Fixation Test
CFU: Colony Forming Units
CIRAD: Centre de coopération internationale en recherche agronomique pour le développement
CVRI: Central Veterinary Research Institute
GALVmed: Global Alliance for Livestock Veterinary Medicines
HT: Hudson and Turner
ILRI: International Livestock Research Institute
IM: Intramuscular
IZS: Instituto Zooprofilactico Sperimantale
KALRO: Kenya Agricultural & Livestock Research Organisation
*Mmm*: *Mycoplasma mycoides* subspecies *mycoides*
OIE: Organization for Animal Health
PBS: Phosphate-buffered saline
PCR-DGGE: Polymerase Chain Reaction - Denaturing Gradient Gel Electrophoresis
PPLO: Pleuropneumonia-like organisms
SC: Subcutaneous
UK: United Kingdom
VSRI: Veterinary Science Research Institute
